# Advanced Computational Techniques for Plasmonic Metasurfaces in the Detection of Neglected Infectious Diseases

**DOI:** 10.1021/acs.analchem.4c04934

**Published:** 2025-03-27

**Authors:** Felipe M. F. Teixeira, Ary V. R. Portes, Talles E. M. Marques, Yuri H. Isayama, Felipe A. N. de Freitas, Fabiano C. Santana, Aline Mendes da Rocha, Thais F. S. Moraes, Lidia M. Andrade, Alice F. Versiani, Estefânia M. N. Martins, Eduardo A. Cotta, Wagner N. Rodrigues, Ronaldo A. P. Nagem, Flávio G. da Fonseca, Clascidia A. Furtado, Jhonattan C. Ramirez

**Affiliations:** † Departamento de Engenharia Eletrônica, Universidade Federal de Minas Gerais, Belo Horizonte 31270-901, Brazil; ‡ Programa de Pós-Graduação em Engenharia Elétrica, Universidade Federal de Minas Gerais, Belo Horizonte 31270-901, Brazil; ¶ Departamento de Física, Universidade Federal de Minas Gerais, Belo Horizonte 31270-901, Brazil; § LCPNano, Universidade Federal de Minas Gerais, Belo Horizonte 31270-901, Brazil; ∥ Departamento de Bioquímica e Imunologia, Universidade Federal de Minas Gerais, Belo Horizonte 31270-901, Brazil; ⊥ Departamento de Microbiologia, Universidade Federal de Minas Gerais, Belo Horizonte 31270-901, Brazil; # Departamento de Morfologia, Universidade Federal de Minas Gerais, Belo Horizonte 31270-901, Brazil; @ Centro de Desenvolvimento da Tecnologia Nuclear, Belo Horizonte 31270-901, Brazil; ∇ Departamento de Física, Universidade Federal do Amazonas, Manaus 69067-005, Brazil

## Introduction

Infectious diseases, caused by pathogenic agents, pose severe risks to both human and animal health, disrupting normal physiological functions and imposing an enormous burden on public health systems globally.^1^ This burden is particularly acute in developing countries, where the lack of resources exacerbates the impacts of these diseases.^2^ Notable examples such as influenza, tuberculosis, leishmaniasis, paracoccidioidomycosis, and malaria claim millions of lives annually. The rapid spread and high mortality of certain diseases, such as COVID-19 and Ebola, have heightened global awareness, while others, like HIV/AIDS and Dengue, present ongoing challenges due to the protracted and expensive development of effective treatments and vaccines.^3,4^

Accurate, early, and accessible detection of infectious agents is paramount for effective patient care and informed public health strategies. Existing diagnostic tools, including molecular techniques such as RT-PCR and RT-LAMP, and innovative approaches like CRISPR-Cas, complement immunological methods such as ELISA and lateral flow assays.^5^ Despite their utility, these methods often face limitations such as extended processing times, high costs, and susceptibility to diagnostic errors, creating a pressing need for more accurate, cost-effective, and point-of-care solutions.^6^

Nanotechnology has emerged as a transformative approach in biomedical diagnostics, particularly for infectious diseases.^7,8^ Its advancements offer unparalleled improvements in sensitivity, processing speed, and device miniaturization compared to traditional techniques.^9^ For instance, gold nanoparticles have proven instrumental in diagnostic applications, enhancing detection sensitivity through their unique optical properties.^10,11^ Functionalization of nanomaterials with disease-specific antigens further enables early and precise detection, making them valuable tools in combating infectious diseases.^8^

Despite the promise of nanotechnology, many of its applications, such as platforms utilizing spherical gold nanoparticles or nanorods, remain largely confined to research settings and have not transitioned to validated commercial products.^12^ Key challenges include inconsistent nanoparticle distribution in aqueous media, variability in plasmonic coupling, and issues like analyte saturation on particle surfaces, all of which compromise diagnostic reliability.^13^

To address these challenges, biosensing platforms based on metasurfaces have emerged as a promising alternative.^14^ These metasurface-based systems offer the potential for superior sensitivity, selectivity, and robustness, overcoming the barriers that limit the adoption of nanoparticle-based diagnostics. Recent innovations in localized surface plasmon resonance (LSPR) technology, coupled with chip-scale metasurfaces and microfluidic integration, have further advanced the capabilities of these platforms. Such systems hold the potential to mitigate nanoparticle-related uncertainties in aqueous environments, paving the way for efficient, accurate, and commercially viable biosensors.^6^

This tutorial provides a comprehensive overview of the development and implementation of metasurface-based biosensing platforms for detecting neglected infectious diseases, focusing on their design, functionality, and integration into diagnostic workflows. By bridging the gap between cutting-edge nanotechnology and its practical applications, it empowers readers to appreciate their transformative role in analytical chemistry and their potential for impactful global health solutions.

## Plasmonic Metasurfaces

Plasmonic metasurfaces, composed of precisely engineered metallic nanostructures, have revolutionized the field of optical sensing and nanophotonics by leveraging the unique interaction of light with conduction electrons in metals. These metasurfaces exploit localized surface plasmon resonance (LSPR) and propagating surface plasmon resonance (PSPR) phenomena to achieve exceptional optical field confinement and environmental sensitivity.^15^ The periodic arrangement of metallic nanostructures enables hybrid plasmonic modes, where the interplay between localized plasmons and diffraction effects enhances the resonance characteristics, including narrowing and redshifting of the LSPR peaks compared to isolated nanoparticles.^16−19^

This coupling of plasmonic modes with geometric resonances, driven by periodicity and surrounding dielectric properties, underpins the design of highly sensitive devices for biosensing, structural characterization, and quantum emitter enhancement.^20^ Theoretical insights, such as Bloch’s theorem, describe the in-plane wavevector relationships in periodic arrays, providing predictive power for mode behavior under varying incident angles and substrate properties.^21^ These properties are formally expressed in eq 1 and are central to optimizing metasurface configurations for specific applications.^22,23^
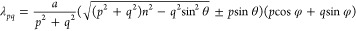
1

**Figure 1:**
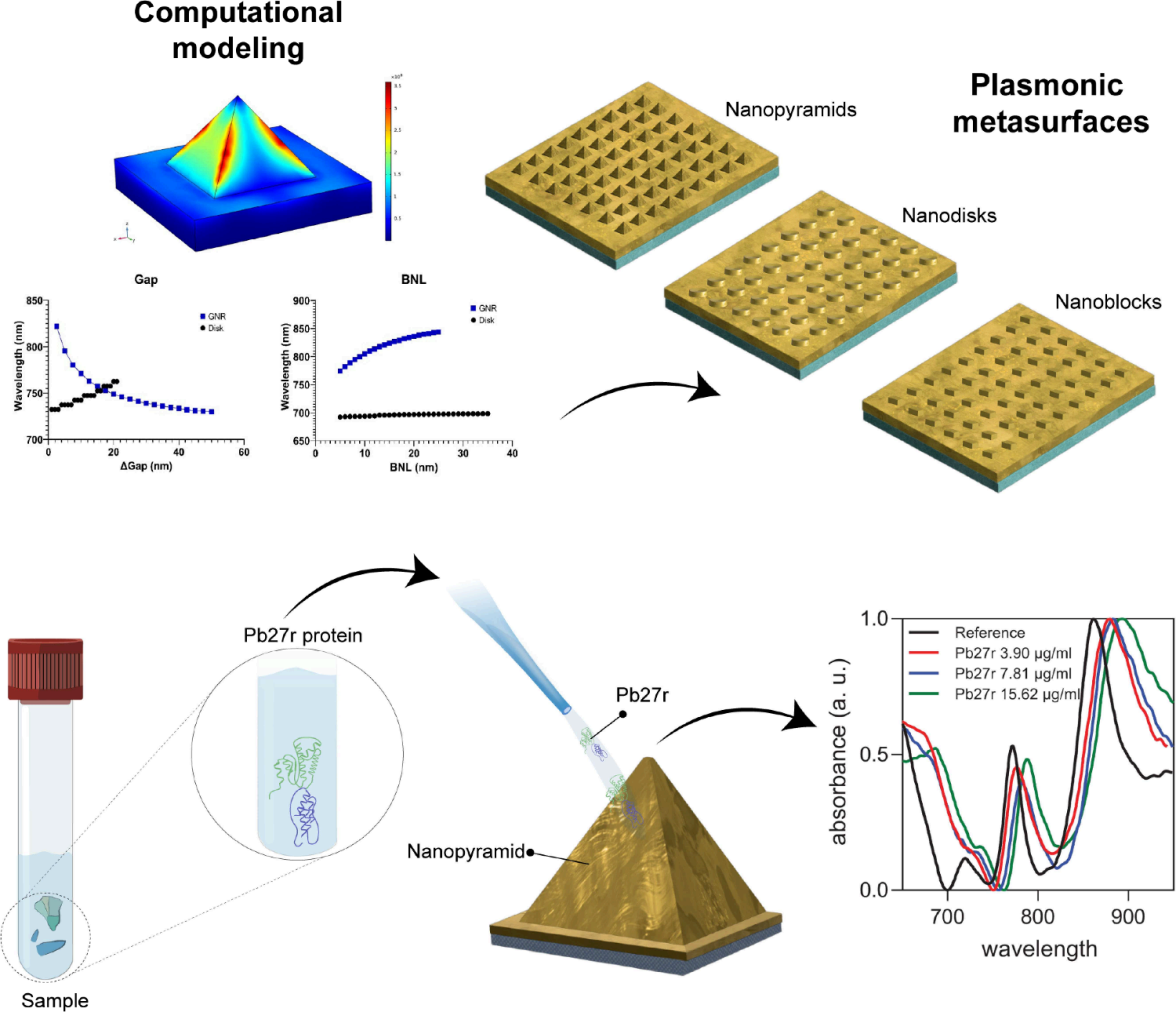
Illustration of the complete workflow for modeling plasmonic metasurfaces, from design and optimization to their integration into biodetection systems for enhanced diagnostic applications.^25^ Reprinted in part with permission from ref (25). Copyright 2024 ACS Publications.

Recent advances in plasmonic metasurface design, including nanopyramids and nanodisks, have demonstrated superior sensitivity and stability under experimental conditions.^24^ For example, as shown in Figure 1, these structures exhibit tunable resonance shifts in response to variations in gap size and biological nanolayer thickness, highlighting their adaptability for biosensing applications.^24^ The electric field localization at critical points, such as pyramid edges or nanodisk gaps, further amplifies their utility in detecting subtle changes in the refractive index or molecular adsorption.^25^

The unique ability of plasmonic metasurfaces to combine sharp spectral features with strong field enhancement enables precise control over light–matter interactions.^26^ By exploiting coherent superpositions of incident and scattered fields, these metasurfaces achieve collective electromagnetic resonances highly responsive to geometric and environmental factors.^26,27^ Additionally, in metal-dielectric configurations, the tunneling of surface plasmons enhances mode coupling across multilayered structures, resulting in tailored energy modes that are pivotal for advanced sensing and energy applications.^28,29^

## Modeling Plasmonic Metasurfaces

The morphology of nanoparticles profoundly influences their plasmonic properties, as the geometry dictates the distribution and intensity of localized surface plasmon resonance (LSPR). For instance, sharp vertices in triangular or star-shaped nanoparticles concentrate electric fields, enhancing sensitivity to environmental changes.^27^ However, the intricate fabrication requirements for sharp-edged designs often pose significant challenges. Rounded nanoparticles, while easier to manufacture, may exhibit reduced sensitivity due to less pronounced field enhancement effects.^30^ Similarly, interparticle spacing critically determines plasmonic coupling: tighter gaps can amplify electromagnetic interactions, whereas increased spacing dampens these effects. Fabricating metasurfaces with precise interparticle separations remains a technological hurdle, particularly in systems involving biofunctionalization, where distance variations alter the resonance.^10^ For fixed substrates, considerations such as nanoparticle spacing, substrate thickness, and composition are paramount, as they collectively influence the overall optical response.

Efficient light coupling into plasmonic systems is integral to the design of LSPR biosensors. The choice of substrate material plays a critical role in optimizing plasmonic metasurfaces for specific applications, such as disease detection or environmental sensing. Noble metals, particularly gold (Au) and silver (Ag), dominate plasmonic applications due to their chemical stability and superior resistance to oxidation.^31^ On planar substrates, these metals support surface plasmon polaritons (SPPs), which can be described mathematically as^32^
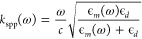
2where *k*_spp_ is the SPP wavevector, ω the electromagnetic frequency, *c* the speed of light, and ϵ_*m*_(ω), ϵ_*d*_ the dielectric constants of the metal and surrounding medium, respectively.

Patterned metasurfaces extend the versatility of plasmonic platforms by supporting multiple resonance modes observable in reflection spectra.^33^ A high-quality factor (Q-factor), indicative of narrow resonance line widths and efficient energy transfer, is crucial for optimizing LSPR-based applications.^34^ The Q-factor is expressed as
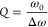
3where ω_0_ is the resonant angular frequency, and Δω is the bandwidth of the resonance. To achieve high Q-factors, metasurface geometries are frequently refined using advanced numerical simulations and optimization techniques, such as shape optimization, inverse design, machine learning algorithms, among others.

Dielectric spacers have also garnered attention for their role in enhancing gap surface plasmon excitation, which yields higher field confinement and absorption efficiency.^35^ The integration of semiconductors into plasmonic systems introduces exciton-polaritons, which further bolster light–matter interactions and may substantially improve biosensor sensitivity.^36^ These materials’ tunable optical properties allow precise customization of plasmonic responses for specific sensing wavelengths.^25^

In addition, two-dimensional (2D) materials, such as graphene, molybdenum disulfide (*MoS*_2_), and black phosphorus, have recently emerged as transformative options for enhancing plasmonic biosensing.^37−39^ Graphene, with its exceptional carrier mobility and adjustable optical conductivity, is particularly noteworthy for improving plasmonic responses.^40^ Furthermore, the atomic-scale thickness of 2D materials facilitates extreme electromagnetic field confinement, significantly enhancing detection capabilities.

These advancements in materials and substrate design lay the foundation for more sophisticated plasmonic metasurfaces. To fully harness these opportunities, employing advanced optimization techniques is imperative to fine-tune the interplay of geometry, material properties, and light–matter interactions. A detailed description of the computational and numerical methods employed in this modeling process, as well as the criteria for selecting the most suitable figure of merit, is provided in the Supporting Information.

## Advanced Optimization Techniques

Optimization of plasmonic metasurfaces has progressed beyond traditional heuristic approaches, leveraging methods such as inverse design and artificial neural networks (ANNs). These strategies have significantly enhanced the design precision and functional versatility required for next-generation biosensors, which demand the integration of vast data sets and sophisticated algorithms to achieve unprecedented performance metrics.^34,41^

**Figure 2:**
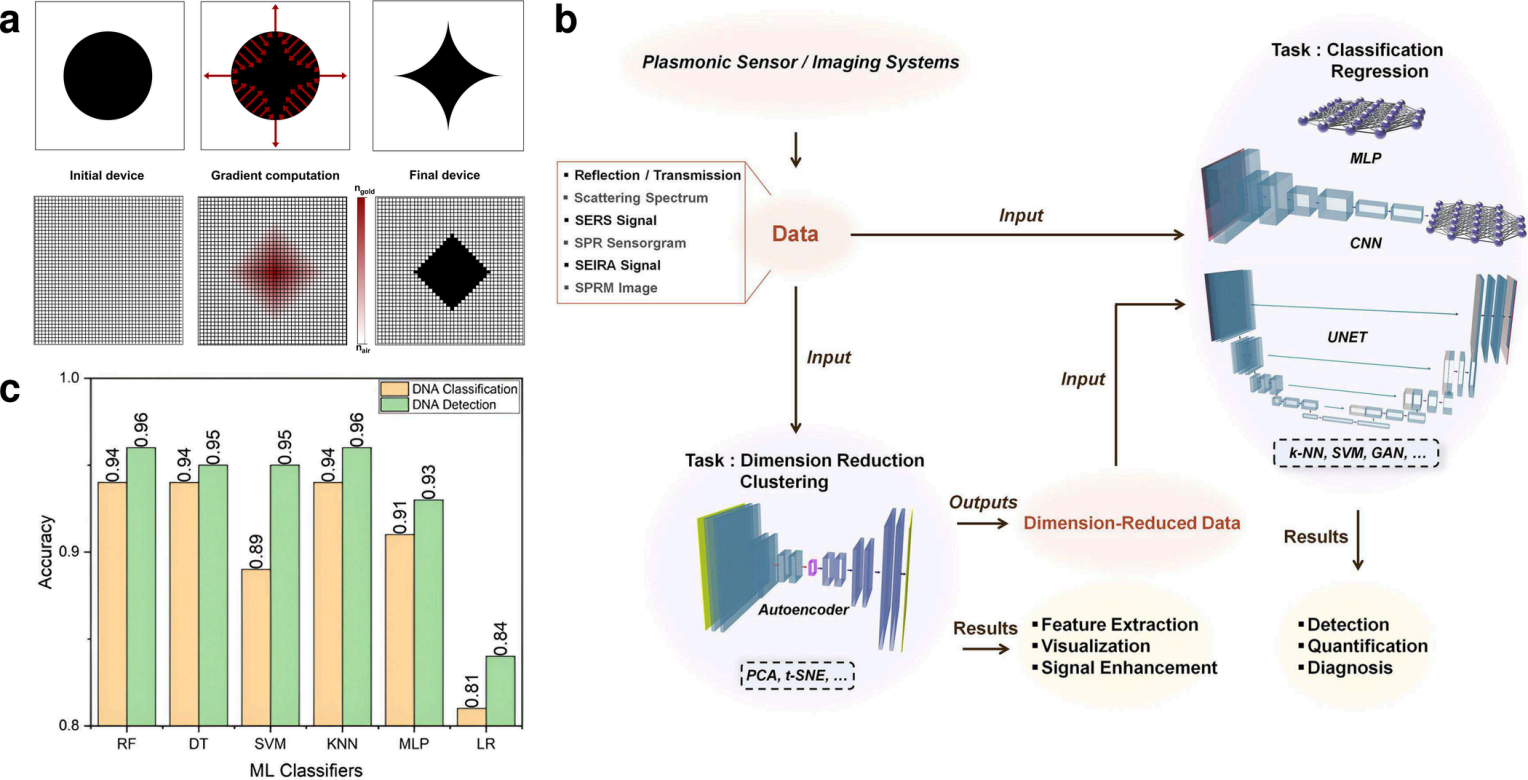
Advanced techniques for designing plasmonic devices. (a) Procedure for shape (top) and topology (bottom) optimization. (b) Machine learning pipeline for analysis of data measured from plasmonic devices.^46^ (c) Prediction accuracy of DNA classification and detection in a plasmonic biosensor for different classifiers. Reprinted in part with permission from ref (46). Copyright 2022 Elsevier.

First, it is worth noting, that inverse design methods start with a targeted plasmonic response, employing adjoint optimization to iteratively refine design parameters. This approach calculates detailed gradient information, enabling efficient exploration of the design space and circumventing suboptimal configurations that plague forward-only methods.^34,42,43^ For instance, topology optimization facilitates complex material redistribution, while shape optimization allows the deformation of classical shapes to create nonintuitive nanostructures, as shown in Figure 2a.^44^ Such techniques have demonstrated transformative applications in sensing, where precise field enhancement is critical.^34,45^

In the same way, ANNs have emerged as indispensable tools in metasurface design by serving as surrogate models for computationally expensive simulations.^47^ Once trained, ANNs predict optical responses with remarkable speed, facilitating the optimization process of these devices.

These tools have become indispensable as the growing complexity of data generated in nanophotonics demands the integration of AI algorithms. Future biosensor platforms will rely on these advanced computational methods to interpret and optimize multilayered data sets in real time, ensuring accurate and efficient performance across diverse applications.^41^ For example, multitask deep-learning models have been shown to efficiently address forward and inverse design problems in chiral plasmonic metamaterials, significantly accelerating the iterative optimization process while maintaining high precision.^45^ These models utilize joint-learning frameworks to simultaneously predict optical responses and retrieve corresponding geometric parameters, illustrating a direct path toward automated design pipelines.^45^

By leveraging these advanced optimization techniques, researchers can tailor plasmonic devices to specific biosensing challenges, such as enhancing the sensitivity and selectivity of molecular detection. Figure 2b illustrates ANN-driven workflows, demonstrating the procedure to obtain data from numerical simulations to train a neural network, that will provide predictions on the best parameters for a plasmonic device. Figure 2c demonstrates the results for the detection and classification of DNA in a plasmonic biosensor using different ANN.^48^ The results demonstrate over that three over the five networks obtained 90% in both tasks, demonstrating the effectiveness of these methods in providing predictions. Such innovations are pivotal for applications like pathogen detection, cancer biomarker sensing, and drug monitoring, where rapid, accurate, and scalable solutions are critical.^41,49^

These advancements not only highlight the transformative potential of AI-driven design in nanophotonics but also underscore the essential role of machine learning in bridging the gap between theoretical optimization and practical applications in analytical chemistry. As biosensor technologies continue to evolve, embracing AI will be paramount in meeting the demands of precision medicine and public health surveillance.^41^

## Manufacturing Process

Manufacturing plasmonic metasurfaces based on nanoparticle lattices is a critical process for developing advanced biosensing devices. Various fabrication techniques have been developed for constructing these devices, with the choice of method depending on specific parameters such as the desired resolution, dimensional constraints, and application-specific requirements. Table 1 provides a comprehensive overview of the key fabrication methods for metasurfaces, highlighting their respective advantages and limitations.

**Table 1:** Comparison of Manufacturing Techniques for Metasurfaces

Technique	Description	Advantages	Disadvantages	Resolution	Size Limit	Scalability	Consistency	Cost-Effectiveness	Mass Production	References
Electron Beam Lithography (EBL)	High-resolution patterning using focused electron beams.	Unparalleled resolution (<10 nm), flexible design patterns.	High cost, time-consuming, limited throughput.	<10 nm	∼100 μm^2^	Limited	High	Low	No	(18, 19, 23)
Focused Ion Beam (FIB)	Uses ion beams for direct nanoscale sculpting and material deposition.	Direct and maskless process, versatile for various materials.	Slow and costly, potential material damage due to ion implantation.	∼5 nm	∼100 μm^2^	Limited	Medium	Low	No	(18, 19)
Nanoimprint Lithography (NIL)	Transfers nanoscale patterns onto substrates using molds.	High throughput, cost-effective for mass production, excellent replication fidelity.	Requires durable molds, limited design flexibility.	∼10 nm	∼10 cm^2^	High	High	High	Yes	(19, 23)
Photolithography	Transfers patterns using light and masks.	Mature, integrates with industrial processes.	Resolution limited by light wavelength.	∼50 nm (EUV)	>1 m^2^	High	High	High	Yes	(18, 23)
Colloidal Self-Assembly	Uses self-organization of nanoparticles to create periodic structures.	Low cost, scalable, environmentally friendly.	Limited control over defect density and arrangement.	∼50 nm (pitch)	>1 cm^2^	High	Medium	High	Yes	(18, 23)
Layer-by-Layer Assembly	Sequential deposition of materials to build metasurfaces.	Precise control over material composition.	Labor-intensive, slow, low throughput.	1 nm (thickness)	>1 m^2^	Low	High	Low	No	(19, 23)

Electron Beam Lithography (E-Beam) and Focused Ion Beam (FIB) milling are two fabrication techniques widely employed for creating plasmonic metasurfaces,^50^ each offering distinct advantages depending on application requirements. E-Beam enables high-resolution patterning over larger areas, making it ideal for fabricating uniform nanoparticle arrays.^51,52^ In contrast, FIB excels at crafting intricate or three-dimensional structures, though its slower speed and smaller working area make it more suited for specialized designs.^50,53^ A comparative summary of these methods and others is presented in Table 1, providing context for their roles in advancing scalable and precise metasurface fabrication.

Electron Beam Lithography (E-Beam) involves a multistep process where a resist layer (e.g., poly(methyl methacrylate), PMMA) is spin-coated onto a clean substrate, such as silicon.^54^ A high-resolution electron beam is then used to define nanoparticle lattice patterns on the resist, followed by metal deposition and a lift-off process, resulting in highly precise arrays of metallic nanoparticles.^25^ This technique is particularly effective for creating large-area, uniform structures with sub-10 nm resolution.^55^

Focused Ion Beam (FIB) Milling, in contrast, directly etches the nanoparticle lattice into the substrate without requiring a resist layer.^50^ This approach offers exceptional flexibility and precision for fabricating complex or three-dimensional structures.^50,53^ However, its slower processing speed and higher cost make it more suitable for small-scale applications requiring intricate designs.^55^

**Figure 3:**
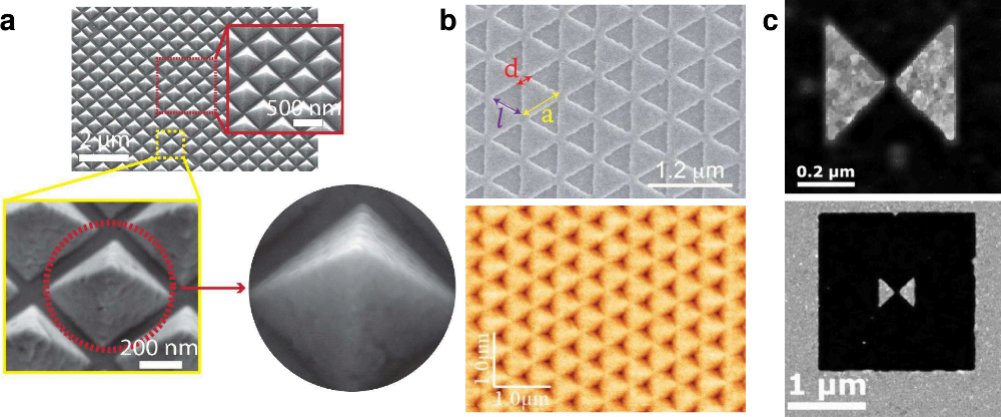
(a) Example of a fabricated pyramid array utilizing e-beam lithography.^25^ (b) Example of pyramidal nanoholes fabricated with e-beam lithography observed with scanning electron microscopy (SEM) and atomic force microscopy (AFM).^51^ (c) Example of nanoantennae fabricated with FIB lithography observed with scanning transmission electron microscopy (STEM) coupled with electron energy loss spectroscopy (EELS).^53^ Reproduced with permission from ref (25). Copyright 2024 ACS Publications. Reproduced with permission from ref (51). Copyright 2021 ACS Publications. Reproduced with permission from ref (53). Copyright 2024 ACS Publications.

While both techniques provide the precision needed for plasmonic metasurfaces, E-Beam is better suited for large-area uniformity, whereas FIB excels in producing detailed, small-scale structures. Figure 3 showcases devices with varying shapes fabricated using these methods, demonstrating their ability to achieve high-quality finishes and precise structural definition.

Template-based fabrication is an alternative approach that enhances scalability while maintaining high precision.^25,56^ Using a master template created by E-Beam or FIB, this method allows for the production of multiple identical metasurfaces.^25^ The template can undergo further processing via wet or dry etching to achieve the desired nanostructure geometry. For instance, wet etching of a 100-oriented silicon wafer with KOH produces nanopyramid arrays.^25^ After etching, a metal layer (e.g., gold) can be deposited and detached from the substrate under controlled conditions, leaving the template reusable.^25^

Figure 3a presents the fabricated array of pyramids using the template-based process with e-beam. These devices present consistent size distributions and alignment with target dimensions, as shown by the figure. Figure 3b evidence the high uniformity and regularity of the metallic nanoholes over the whole patterned area using e-beam.^22,51^ Moreover, Figure 3c showcases the FIB fabrication precision, producing nanoantennae that do not exhibit residual grains.^27,53^

In this sense, some advantages of template-based fabrication are perceived as can be seen below:

- Scalability: Reusability of the master template enables efficient production of multiple devices.
- Cost-Effectiveness: Minimizes the need for repetitive, time-intensive lithography steps.
- Consistency: Ensures uniformity critical for biosensing applications requiring reproducible performance.

Integrating template-based fabrication with E-Beam lithography and FIB milling enables researchers to balance precision, scalability, and cost-effectiveness in producing plasmonic metasurfaces.

## Nanoplatforms for Detection of Infectious Diseases

Metasurfaces, engineered nanoplatforms with unique physicochemical properties, have emerged as versatile tools in advanced diagnostics due to their exceptional sensitivity and specificity. Metallic nanoplatforms, particularly gold nanoparticles, are of particular interest for their ability to harness localized surface plasmon resonance (LSPR). This phenomenon, driven by collective electron oscillations at the nanoparticle surface, generates evanescent fields that extend into the surrounding medium. Such sensitivity to minute environmental changes enables the precise detection of low-abundance biomarkers, offering significant advantages over conventional diagnostic methods. The ability to chemically functionalize these nanoplatforms for targeted interaction with biological molecules further enhances their utility in analytical applications.^57,58^

The functionalization of nanoplatforms is most commonly observed in nanorods and nanospheres due to their well-established preparation protocols and versatility. Thus, the following sections will focus on the functionalization procedures for these nanoparticle geometries. However, it is important to note that nanoparticle aggregation significantly increases the complexity of these processes, adding additional challenges during implementation. Despite these complexities, the functionalization strategies outlined here are equally applicable to plasmonic metasurfaces, which share similar biochemical challenges and technical hurdles at the nanometric scale. The core biochemical processes, including surface modification, ligand attachment, and stability optimization, remain fundamentally consistent across both nanoparticle platforms and metasurfaces, demanding high precision and control to ensure reliable functionality and reproducibility in biosensing applications.

## Functionalization of Gold Nanorods with Biomolecules

Functionalizing gold nanorods (GNRs) allows its specificity and sensitivity for disease detection, making them valuable tools in diagnostic assays. GNRs can be functionalized with biomolecules such as proteins, peptides, antibodies, and aptamers using covalent or noncovalent conjugation.^8,25,59−62^ The chosen method affects the bioassay’s precision, accuracy, and stability and, thus, the reliability of analytical results. Covalent conjugation involves creating stable chemical bonds between the nanomaterial and the biomolecule, achieved via cross-linking agents through diimide-activated amidation,^63−65^ click chemistry,^66,67^ or surface group modifications.^68^ Noncovalent adsorption, while simpler, relies on weaker interactions such as electrostatic and van der Waals forces, making it less stable than covalent methods.^69−71^

**Figure 4:**
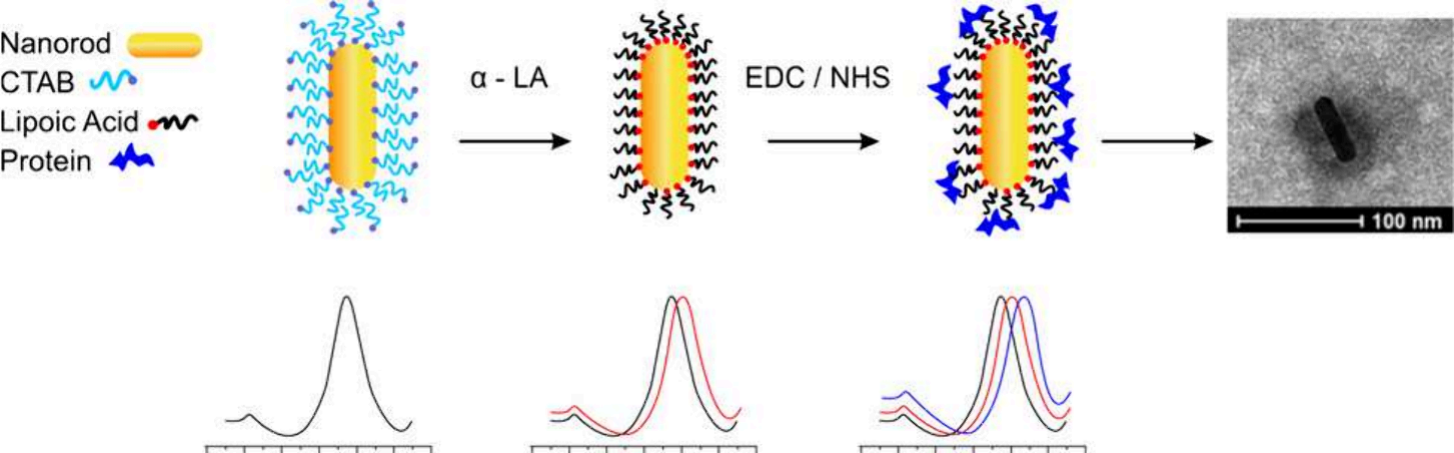
Schematic representation of the functionalization of gold nanorods with biomolecules. (a) Gold nanorod synthesized by the seed-mediated growth method, coated with CTAB; (b) Gold nanorod coated with alpha-lipoic acid (LA); (c) Gold nanorod coated with LA and functionalized with a biomolecule. The sequence shown in this figure illustrates the functionalization process previously described. (Created in CorelDRAW Graphics Suite, by Raphael Gomes de Paula (2024), *Used with permission*).

Typically, GNR synthesis involves seed-mediated growth with CTAB as a growth-directing agent. An initial functionalization step includes replacing excess CTAB with cationic surfactants like α-lipoic acid (α-LA), which has a strong affinity for gold surfaces due to its sulfur-containing functional groups. This creates stable thiolate-gold bonds and facilitates protein attachment via covalent bonding through α-LA’s carboxylic acid groups.^8,60,62^ The amidation reaction, facilitated by cross-linking agents like EDC (1-ethyl-3-(3-(dimethylamino)propyl) carbodiimide) and NHS (*N*-hydroxysuccinimide), is commonly used for covalently bonding proteins to GNRs. EDC activates the carboxyl groups on α-LA, while NHS stabilizes these intermediates, enabling efficient amide bond formation with protein amine groups.^63,64^ Protein attachment efficiency can be assessed through spectroscopy, microscopy, or colorimetric assays.^72^ Surface blocking, using agents like thiolated polyethylene glycol (PEG-SH), is a crucial step after protein attachment. It prevents nonspecific binding and reduces background noise, ensuring the accuracy and reliability of the biosensor results.^73^Figure 4 provides a step-by-step depiction of the biofunctionalization process, illustrating the transformation of nanoparticles at each stage. The graphical representation complements the detailed description, offering a clear visual guide to the progression and key changes occurring during the functionalization. At the end of the process, the gold nanorod functionalized with the specific protein can be visualized by transmission electron microscopy as caped with an amorphous substance.

According to the detailed physical-chemical characterization process described in the Supporting Information, a comprehensive evaluation of the functionalization process confirms the successful attachment of biomolecules, the preservation of colloidal stability, and the suitability of the nanomaterials for biosensor applications. This assessment underscores the reliability of the functionalization strategy in achieving the necessary biochemical and physical properties for effective biosensing.

## Optimization and Validation of the Fabrication Process to Enhance the Assay Diagnostic Performance

Several techniques exist for developing reliable nanosensors as diagnostic tools. Following a successful proof-of-concept demonstrating target analyte detection, optimization is critical to refine assay performance by adjusting key chemical, physical, and biological parameters.^74,75^ Reference samples, including verified positive and negative controls or spiked samples with defined analyte concentrations, are essential during this process. Cross-reactivity must also be assessed, and assay thresholds established from experimental data.^8,76^ Key considerations for assay development and validation include:

1. Assay Purpose: Defining the assay’s intended purpose is crucial, as multiple factors can influence the results. Host factors (age, sex, nutrition, pregnancy, immunological responsiveness), as well as disease incidence and prevalence, can affect assay sensitivity and specificity. The assay may be qualitative or quantitative, confirmatory, or used for screening, disease prevalence estimation, or surveillance. A single assay can be optimized and validated for multiple purposes by fine-tuning diagnostic performance metrics, including sensitivity, specificity, and predictive values;
2. Sample Type: The matrix in which the analyte is found must be considered when developing nanosensors. Different nanosensors may be designed for whole blood, serum, feces, urine, tissue, or environmental samples (e.g., soil, water). Some matrices may contain inhibitors that can interfere with assay performance. Protocols should include specific instructions to avoid erroneous results, and prepurification methods, such as filtration, enzymatic digestion, or centrifugation, are recommended for nonclear samples;
3. Temperature and pH: Both nanosensor development and assay performance are often temperature- and pH-dependent. Proper control of storage and nanoparticle stability at each stage is critical. Before selecting a functionalization process, developers must assess whether the assay conditions (temperature and pH) will affect sample stability;
4. Protein Corona Effect: When exposed to biological samples, a protein corona may spontaneously form on the surface of nanomaterials, potentially altering the nanosensor’s physicochemical properties and interaction with the target analyte.^77,78^ Understanding and mitigating this effect is important to maintain assay accuracy.

**Figure 5:**
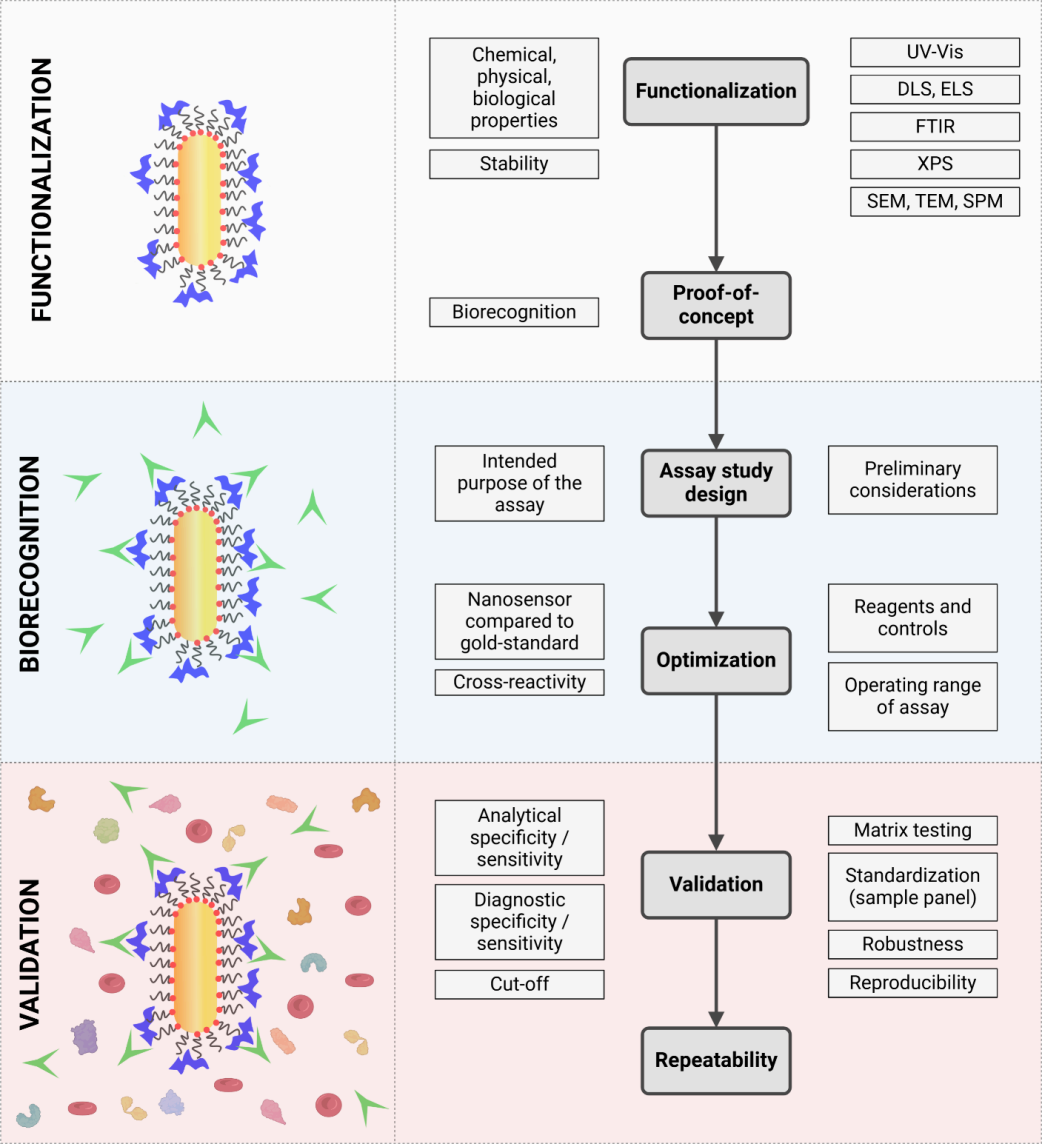
Nanobiosensor assay development process. The three main branches of the development of a gold nanosensor for diagnostic application are particle functionalization, evaluation of proper biorecognition, and validation of the diagnostic tool. The flowchart describes experimental procedures and critical points to consider during the assay assessment. (Created in BioRender. Versiani, A. (2024) BioRender.com/f70w259).

After establishing the conditions outlined above, the nanosensor must undergo validation to confirm its analytical and diagnostic performance for the specific species and sample types intended (Figure 5). This process includes defining the assay’s purpose, optimizing parameters, and evaluating performance metrics such as repeatability, sensitivity, specificity, and reproducibility. Sensitivity (Se) and specificity (Sp) are particularly critical and are often evaluated using the area under the Receiver-Operating Curve (AU-ROC).^79^ Experimental studies during this phase ensure the assay meets the required standards for its intended application.

1. Method design and proof-of-concept: Establishing the assay method requires prior knowledge and planning. Before full validation, the reactivity between functionalized nanoparticles and the target analyte must be determined. Saturation curves and particle stability are assessed;
2. Assay operating range: This range represents the interval of analyte concentrations detectable by the nanosensor method. It helps determine accuracy and precision;
3. Optimization and standardization: Reagent concentrations and assay protocols are fine-tuned using reference samples. These samples help distinguish the target analyte from other molecules and optimize critical assay parameters;
4. Sample matrix validation: Assays must be validated across different sample types (e.g., whole blood vs serum), as matrices may contain inhibitory factors or interfere with measurements, such as LSPR;
5. Assay robustness: This step assesses minor variations, such as changes in pH, temperature, or reagent brands, that could affect assay performance;^80^
6. Calibration of standard reagents: Reference standards, typically characterized by gold-standard assays in peer-reviewed publications, are used for calibration;
7. Test results: Raw data are converted into standardized units or normalized for comparison across different laboratories and settings. Controls ensure normalization and prevent bias;
8. Repeatability: Repeatability measures the consistency of test results within and between runs, whether in the same lab or across different facilities.

## Troubleshooting

Achieving reproducible results in nanoparticle synthesis and functionalization requires meticulous attention to several key factors, including solute quality, colloidal stability, biomolecule attachment efficiency, and nanoplatform storage. This tutorial provides a detailed troubleshooting guide to address common challenges in these areas as follows in the Table 2.

**Table 2:** Troubleshooting

	Problem	Troubleshooting
Quality of solutes	Water with dissolved ions and contaminants. Such ions may react with reagents, causing unintended side reactions or nanoparticle aggregation, disrupting chemical processes, and affecting the properties and performance of the final nanomaterials.	Prioritize the utilization of ultrapure or deionized water with high resistivity and low conductivity.
		Proper pH control, as pH deviations can lead to aggregation or alteration of nanomaterial characteristics.
	Microbial contamination. It can affect any steps of the sensor construction or validation, heavily impacting on the device outcome.	Solutes should be filtered and sterilized. A proper quality control procedure for all solutes utilized must be implemented and reported.
	Endotoxin contamination can interfere or misidentify biological effects on nanostructures.	Use of endotoxin-free water. Implementation of endotoxin quantification and removal process throughout the functionalization steps to maintain quality control.
	Chemical contamination.	Every solution must be prepared and stored properly. All containers, plastic or glassware, must be thoroughly cleaned before and after usage. Additionally, all glassware should be cleaned with aqua regia and rinsed with ultrapure water to avoid contaminants before reuse.
Colloidal Stability of Nanoparticles	Issues regarding nanoparticle aggregation.	Usually related to solute contamination, pH, and/or glassware properly clean.
		The choice of surfactant is critical and must be included as a preliminary consideration.
		Mechanical forces applied during bath sonication and centrifugation must be carefully controlled. Excessive force can lead to aggregation or size alterations, while insufficient force may result in incomplete dispersion. Optimizing these parameters helps maintain stable colloidal dispersions and prevents aggregation.
Biomolecule attachment efficiency	Issues with bidding affinity.	Efficient biomolecule attachment to nanoparticles in an aqueous medium depends on understanding the biomolecules’ characteristics, such as isoelectric point (pI), hydrophobicity, hydrophilicity, size, shape, and charge. Aligning the pH of the solution with the biomolecule’s pI can enhance electrostatic interactions, while balancing hydrophobic and hydrophilic properties affects binding affinity.
		The biomolecule’s size and shape should correspond to the nanoparticle surface for optimal attachment, and compatibility between the biomolecule’s charge and the nanoparticle surface charge is essential for stable binding
Nanoplatform storage optimization.	Long-term stability of nanoplatforms.	Solutions must be stored in amber glass containers to protect from light.
		Polystyrene flasks should be avoided, as they may cause contamination.
		Metal-free, thoroughly cleaned glassware should be used for storage.
		Ensure the correct storage temperature is critical for maintaining the stability and functionality of the nanoplatforms.

## Optical Characterization of Biosensors Based on Plasmonic Metasurfaces

**Figure 6:**
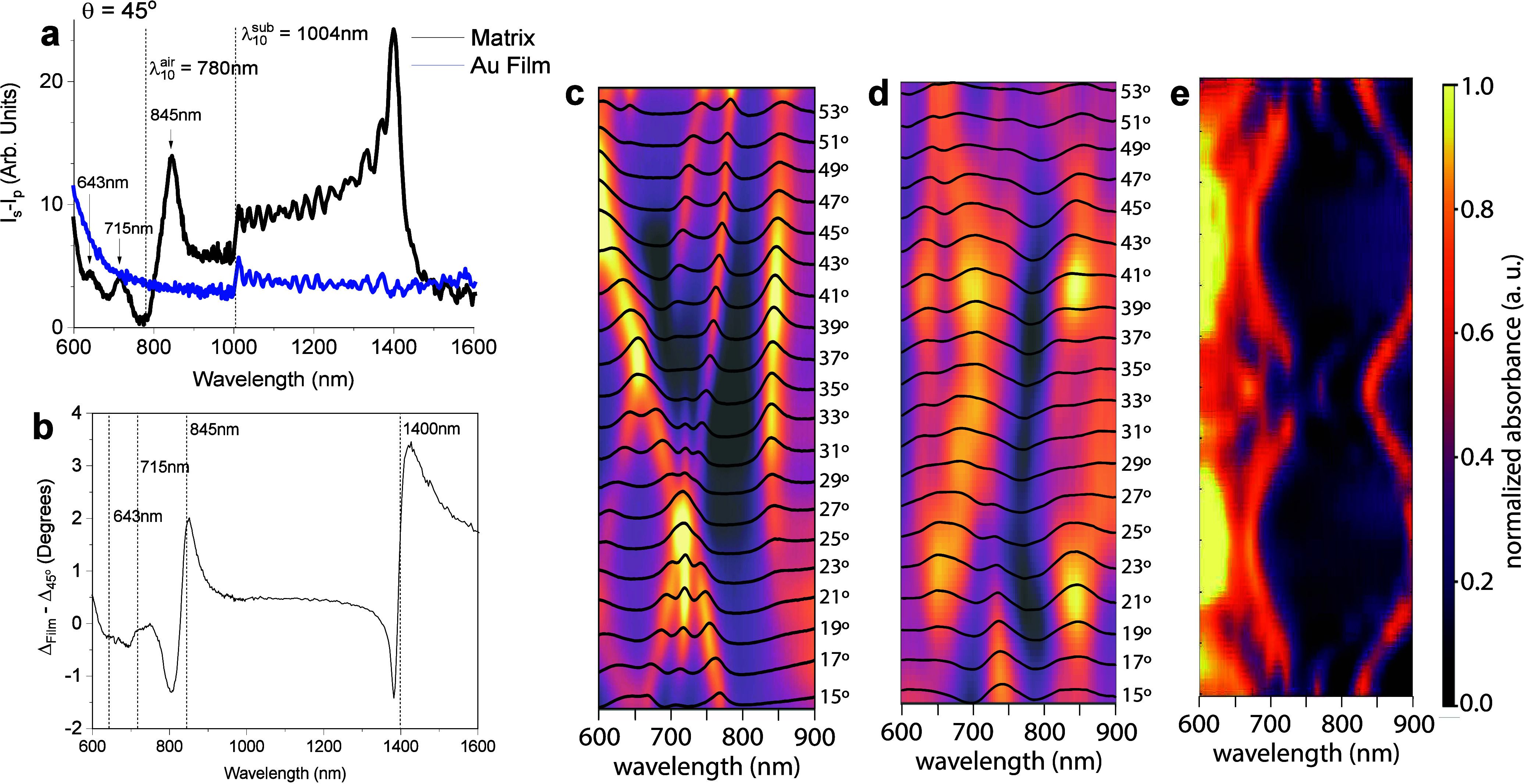
(a) Difference in reflected intensity between s- and p-polarizations (*I*_*S*_ – *I*_*P*_) for a nanopyramid array with a base of 388 nm, height 274 nm, and spacing of 69 nm. The inset shows a top-view electron microscopy image of the nanostructure. Vertical dashed lines indicate the λ_10_^*air*^ and λ_10_^*sub*^ diffraction edges at φ = 0. Arrows mark the spectral positions of the top mode (845 nm), edge modes (715 nm), and base mode (643 nm) scattered by the nanopyramids. (b) Relative phase shift between. (c) Incidence angle variation (15° to 55°) for a metasurface with gold nanopyramids. The resonance peak at 850 nm, selected for its stability, remains nearly constant between 25° and 50°, peaking at 41°, as indicated in the heat map. (d) Measured spectral response of the pyramidal nanoparticle lattice with albumin on its surface. Unlike a, in b, no SPP is observed; only the LSPRs excited in the said structure. This is because the albumin concentration, i.e., 5 mg/mL, saturates the surface, hindering the excitation of the propagating surface plasmons. (e) Measured spectral response of the pyramidal nanoparticle lattice as a function of the azimuthal angle of the incident beam. As shown in this figure, we have a periodic response, which is understandable due to the nature of our pyramidal lattice.^25^ Reprinted in part with permission from ref (25). Copyright 2024 ACS Publications.

The optical properties of plasmonic metasurfaces are central to their performance in biosensing. For a fixed angle of incidence, surface plasmon (SP) excitation results in characteristic reflectance minima and phase shifts near the resonance wavelength (λ_*R*_). These properties, governed by localized surface plasmon resonances (LSPRs) or propagating surface plasmon resonances (PSPRs), vary with the geometry and arrangement of nanostructures. LSPRs typically exhibit broader full-width at half-maximum (fwhm) values (80–100 nm) compared to PSPRs, yielding quality factors (*Q* = λ_*R*_/Δλ) in the range of 10–20. These differences are illustrated in Figure 6a, which highlights the optical response of gold nanopyramids.

## Role of Ellipsometry in Characterization

Ellipsometry is an essential tool for quantifying the optical response of metasurfaces. By measuring amplitude (Ψ) and phase (Δ) shifts in reflected light, ellipsometry provides insights into polarization-dependent effects and resonance modes. Low numerical aperture (*NA*) optics (<0.1) are preferred to ensure spatial coherence over a 2D lattice. For instance, an ellipsometer with *NA* = 0.1 achieves coherence over a 30 × 30 μm spot, enabling precise measurements. Reflectance spectra of nanopyramids (Figure 6a) reveal distinct LSPR absorption modes and diffraction peaks. The prominent peak at λ_*D*_ = 1400 nm, attributed to first-order diffraction, underscores the dual role of nanostructures as plasmonic resonators and optical gratings.

## Geometric and Angular Dependence of Plasmonic Response

The geometry of the metasurface strongly influences its plasmonic response. Pyramidal nanostructures, with their sharp vertices and symmetric design, generate more intense electric fields than planar shapes like nanodiscs or nanoblocks. This enhanced field intensity is advantageous for biosensing, as it improves sensitivity to refractive index changes. As shown in Figure 6c, the absorption spectra were analyzed across incidence angles from 15° to 53°. While a single peak dominated at lower angles, additional peaks appeared with increasing angle, reflecting distinct energy states. The central peak, corresponding to PSPR modes, remained stable, whereas LSPR modes shifted significantly, demonstrating angle-dependent tuning.

## Nanostructured Surfaces for Enhanced Biosensing

The integration of bioreceptors, as those detailed in previous sections, onto the nanostructured surface alters the optical response, enabling specific analyte detection. For example, Figure 6d shows that a 5 mg/mL albumin layer saturates the surface, suppressing PSPR excitation and isolating LSPR modes. This behavior enhances the biosensor’s robustness by maintaining sensitivity across a wide analyte concentration range. Furthermore, the periodic spectral response as a function of azimuthal angle (Figure 6e) underscores the uniformity of the fabricated metasurfaces, a critical factor for reproducibility in biosensing applications.

Despite the complexity of their fabrication, pyramidal nanostructures stand out as an excellent choice for applications demanding high sensitivity and specificity, owing to their unique optical properties and superior performance. The insights discussed in this section highlight how the integration of advanced fabrication techniques with precise optical characterization can pave the way for the development of next-generation biosensing platforms.

## Challenges and Future Directions

### Challenges

Despite the significant advancements in plasmonic metasurfaces, several challenges remain, presenting opportunities for innovation. One key hurdle is achieving stable and specific biofunctionalization of nanostructures for reliable performance in complex biological environments. Issues like protein denaturation, nonspecific binding, and surface degradation continue to impact sensitivity and specificity in diagnostic applications.

Scalability in manufacturing also poses a critical challenge. Techniques like electron beam lithography and focused ion beam milling, while precise, are expensive and time-consuming, limiting their utility for large-scale production. Ensuring batch-to-batch reproducibility is essential for the commercial viability of these technologies.

The integration of these sensors into accessible, portable devices for point-of-care applications remains complex. Durability, user-friendly operation, and the need for compact data analysis tools must be addressed to ensure their effective use in resource-limited settings.

Finally, the regulatory and ethical implications of deploying nanotechnology in healthcare settings require thorough assessment. Concerns regarding the long-term safety, environmental impact, and equitable access to these technologies must be tackled to foster broader adoption.

### Future Directions

Looking ahead, advancements in biofunctionalization techniques hold the promise of overcoming current stability and specificity issues. The use of recombinant proteins, peptides, or engineered surfaces can provide enhanced stability and targeted functionality in diverse biological matrices.

With scalable manufacturing processes and AI-driven design optimization, plasmonic metasurfaces are poised to revolutionize point-of-care diagnostics globally. Innovations in microfabrication, such as template-based methods, can enhance production efficiency and reduce costs, making these technologies more accessible.

Interdisciplinary integration also offers exciting possibilities. Combining plasmonic sensing with electrochemical or fluorescence-based detection methods can enhance diagnostic accuracy and minimize false positives or negatives. Additionally, these multimodal platforms could expand applications beyond healthcare to include environmental monitoring and food safety.

Lastly, collaborative efforts among researchers, policymakers, and industries are essential. Establishing clear regulatory frameworks and accelerating approval processes for plasmonic-based diagnostics will facilitate their deployment in underserved regions, contributing to global health equity and advancing the role of analytical chemistry in solving real-world problems.

By addressing these challenges and leveraging emerging opportunities, plasmonic metasurfaces can realize their full potential as transformative tools in diagnostics, impacting not only healthcare but also policy, sustainability, and beyond.

## Conclusions

In conclusion, this tutorial has presented an in-depth exploration of how plasmonic-based metasurfaces offer promising advancements in detecting neglected infectious diseases. These innovations can enhance diagnostic sensitivity, accuracy, and speed by leveraging surface plasmon–polariton interactions and biofunctionalization techniques, especially in resource-limited settings. The integration of gold nanoparticles and metasurfaces with biomolecules enables precise and rapid detection of disease-specific markers. While traditional diagnostic methods face limitations, the development of scalable, cost-effective, and highly sensitive nanoplatforms represents a transformative approach to overcoming current diagnostic challenges, thus contributing significantly to global health.

## Data Availability

The data that support the findings of this study are available from the corresponding author upon reasonable request.

## Supporting Information Available

Comprehensive details on essential aspects, including the device manufacturing process, the experimental setup, and the preparation of biological samples (PDF)
