# Supplementary material for: Advanced Computational Techniques for Plasmonic Metasurfaces in the Detection of Neglected Infectious Diseases
Source: Anal Chem. 2025 Mar 27;97(13):6813–25. doi: 10.1021/acs.analchem.4c04934 (PMC11983362; doi:10.1021/acs.analchem.4c04934)
Supplement: Supplementary file 1 — ac4c04934_si_001.pdf [file ac4c04934_si_001.pdf]

Supporting Information: Advanced Computational Techniques for Plasmonic Metasurfaces in the Detection of Neglected Infectious Diseases

Felipe M. F. Teixeira,[†,‡] Ary V. R. Portes,[†,‡] Talles E. M. Marques,[†,‡] Yuri H. Isayama,[¶,§] Felipe A. N. de Freitas,[†,‡] Fabiano C. Santana,[¶] Aline Mendes da Rocha,[‖] Thais F. S. Moraes,[⊥] Lidia M. Andrade,[¶,#] Alice F. Versiani,[@] Estefânia M. N. Martins,[@] Eduardo A. Cotta,[△] Wagner N. Rodrigues,[¶] Ronaldo A. P. Nagem,[‖] Flávio G. da Fonseca,[⊥] Clascidia A. Furtado,[@] and Jhonattan C. Ramirez[*,†,‡]

†*Departamento de Engenharia Eletrônica, Universidade Federal de Minas Gerais, Belo Horizonte, Brazil, 31270-901*

‡*Programa de Pós-Graduação em Engenharia Elétrica, Universidade Federal de Minas Gerais, Belo Horizonte, Brazil, 31270-901*

¶*Departamento de Física, Universidade Federal de Minas Gerais, Belo Horizonte, Brazil, 31270-901*

§*LCPNano, Universidade Federal de Minas Gerais, Belo Horizonte, Brazil, 31270-901*

‖*Departamento de Bioquímica e Imunologia, Universidade Federal de Minas Gerais, Belo Horizonte, Brazil, 31270-901*

⊥*Departamento de Microbiologia, Universidade Federal de Minas Gerais, Belo Horizonte, Brazil, 31270-901*

#*Departamento de Morfologia, Universidade Federal de Minas Gerais, Belo Horizonte, Brazil, 31270-901*

@*Centro de Desenvolvimento da Tecnologia Nuclear, Belo Horizonte, Brazil, 31270-901*

Departamento de Física, Universidade Federal do Amazonas, Manaus, Brazil, 69067-005

Contents

Modeling techniques for plasmonic metasurfaces **S3**

Selection of the most suitable numerical method S3

Choosing the adequate figure of merit for design optimization S3

Protein biosynthesis **S4**

Applications in nanotechnology S5

Step 1: Identification of target proteins S5

Step 2: Protein biosynthesis using specific techniques S5

Step 3: Ensuring biomolecule activity throughout the process S6

Physical-chemical characterization of functionalized nanomaterials **S7**

Cost-Effectiveness of Plasmonic Metasurfaces for Biosensing of Infectious Diseases **S8**

References **S9**

Modeling techniques for plasmonic metasurfaces

Selection of the most suitable numerical method

The Finite-Difference Time-Domain (FDTD) and Finite Element Method (FEM) are widely used numerical techniques in plasmonic metasurface design, each offering distinct advantages and challenges.

FDTD:

Advantages: Highly effective for time-dependent phenomena and complex structures. It can also determine spectral responses through Fast Fourier Transform (FFT) with appropriate excitation sources.

Challenges: Computationally expensive for large or finely detailed structures due to structured meshing and significant memory demands in 3D simulations.

FEM:

Advantages: Superior for complex geometries and inhomogeneous materials, with fine control over mesh refinement for localized fields. FEM is computationally efficient when only a single wavelength needs consideration, as it operates in the frequency domain.

Challenges: High memory requirements for 3D simulations, especially in plasmonic devices where fine mesh is necessary. Careful exploitation of structural symmetries and boundary conditions is essential to optimize simulation efficiency.

Choosing the adequate figure of merit for design optimization

One important step in the device design is to determine what will be the metric to measure the sensor's performance. This will depend on the operating physical mechanism of the sensor. For example, if the objective is to design a metasurface suitable for Surface Enhanced Raman Spectroscopy (SERS), the device sensitivity will be directly linked to the intensity of the Raman Scattering phenomena present. Therefore, the main goal of the design is to define a metasurface that enhances the electric field intensity throughout the surface of the device in

the operating wavelength, as such intensity will govern the Raman Scattering. If, in contrast, the sensor works with the principle of Localized Surface Plasmon Resonance (LSPR) and the experiment will employ a broadband light source, one will be interested in the amount of energy that will be coupled to plasmonic resonances as a function of wavelength, that is, its absorption/reflection spectrum.

Protein biosynthesis

Protein biosynthesis plays a critical role in the development of biosensing platforms by enabling the production of specific bioreceptors, such as antibodies and enzymes, that can selectively bind to target biomarkers, thereby enhancing the sensitivity and specificity of disease detection. In addition, it is essential in nanotechnology due to proteins' ability to perform specific biological functions and interact selectively with target molecules. This selectivity is crucial for biosensing platforms, particularly in disease detection.[1,2] However, challenges such as low yield and reproducibility when extracting proteins from native organisms remain. Advances in recombinant DNA technology and bioinformatics have improved the production of target proteins, making them more reliable as bioreceptors in disease-targeting systems.[3,4]

Proteins play a vital role in infectious disease detection, acting as biomarkers and being involved in immune responses. Diagnostic systems can utilize protein interactions, such as antibodies binding to pathogen antigens, for accurate detection. For instance, the presence of specific antibodies can indicate infection, while the detection of parasite proteins confirms active disease.[5–8]

In the context of neglected infectious diseases, the use of protein-functionalized nanosystems enhances diagnostic sensitivity and specificity. By leveraging recombinant protein production, these biosensing platforms can rapidly detect disease-specific markers, supporting better disease management. This approach is a key component in advancing the detection

of neglected infectious diseases, which is the principal aim of this tutorial.

Applications in nanotechnology

Step 1: Identification of target proteins

Identifying target proteins, such as parasite antigens or host antibodies, is essential for developing effective diagnostic tools. The process involves several steps:

1. Isolating the parasite under conditions mimicking host infection;
2. Preparing a homogeneous cell extract from the parasite to obtain a protein-rich fraction;
3. Obtaining serum from infected and non-infected subjects and evaluating it using techniques like immunoprecipitation or ELISA, possibly followed by chromatographic purification;
4. Isolating antigen-antibody pairs using 2D electrophoresis or chromatographic methods;
5. Identifying the amino acid sequences of the target proteins through high-resolution proteomics and bioinformatics.

Step 2: Protein biosynthesis using specific techniques

Once the target protein's amino acid sequence is identified, efficient production is necessary. This process involves:

1. Synthesizing or amplifying the target protein's nucleotide sequence;
2. Inserting the sequence into a plasmid for bacterial expression;
3. Culturing bacteria with the plasmid to produce the target protein under controlled conditions;

4. Purifying the protein by isolating it from other cellular components using methods like cell disruption, chromatography, and molecular filtration;

5. Confirming the protein's identity and quantity using techniques like electrophoresis, spectrophotometry, and mass spectrometry.[9–11]

Step 3: Ensuring biomolecule activity throughout the process

Maintaining protein activity is critical for their function as bioreceptors. Key considerations include:

1. Keeping proteins in mild, physiological-like conditions, using appropriate buffers and avoiding denaturing agents and high temperatures;[12]

2. Monitoring protein folding and aggregation using techniques such as zeta potential, Dynamic Light Scattering (DLS), Circular Dichroism (CD), Differential Scanning Calorimetry (DSC), and Thermal Shift Assay (TSA) to assess the protein's structural integrity;[13–16]

3. Evaluating protein-ligand binding to ensure effective interaction between the bioreceptor and the analyte, using methods like ELISA,[17] fluorimetry,[18] and Isothermal Titration Calorimetry (ITC)[19] to determine binding strength and stability.

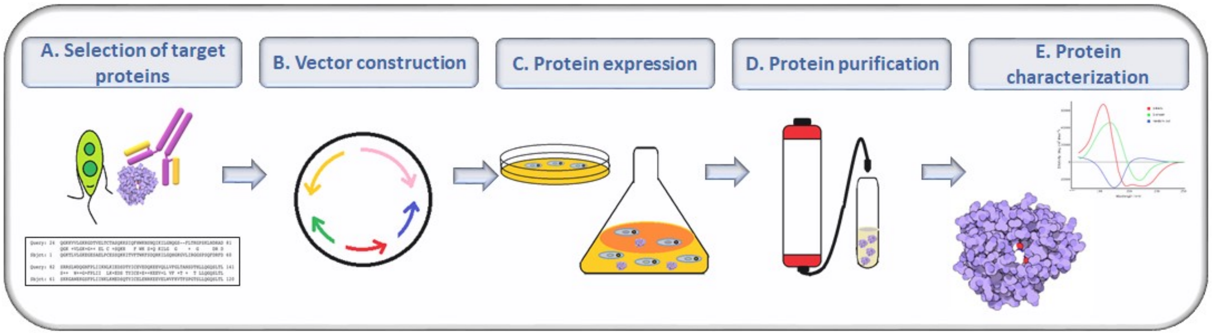

Figure S1: Schematic of protein biosynthesis process relevant to nanotechnological integration. (a) Target proteins are identified by selecting antigens reactive to specific antibodies from parasite-infected organisms. The antigen-antibody complex is used to identify the target protein and its sequence. (b) The target protein gene sequence is inserted into an expression vector. (c) The host organism is transformed with the expression vector and cultured for protein expression. (d) The target protein is purified from cell lysate using chromatography. (e) Protein identity, purity, structure, and stability are confirmed through characterization methods, with activity validated by ligand-binding tests or immunoassays.

Building on the advancements in protein biosynthesis presented by Fig. S1, these biologically functional proteins can now be effectively integrated into nanoparticle-based systems to create and evaluate highly sensitive and specific nanoplatforms for detecting infectious diseases. Protein-functionalized nanoparticles, such as those coated with antibodies or enzymes, can target and bind to specific disease markers, allowing for rapid and precise diagnostics. These nanoplatforms capitalize on enhanced protein production techniques, enabling the development of reliable, scalable, and cost-effective biosensors, which are especially valuable in the early detection of infectious diseases, including neglected tropical diseases.

Physical-chemical characterization of functionalized nanomaterials

Understanding 1) the physicochemical properties of AuNPs and their hybrids, 2) the efficacy and stability of the functionalization process, 3) the biomolecular distribution, coverage, and orientation, and 4) the interactions between constituents, the medium, and the target is essential for directing and controlling biological responses. Achieving this requires com-

prehensive physicochemical characterization using both classical and advanced techniques, such as:

1. Dynamic Light Scattering (DLS): Estimates the hydrodynamic radius to assess the stability and dispersion of the nanomaterials;

2. Electrophoretic Light Scattering (ELS): Measures zeta potential to predict colloidal stability and prevent aggregation;

3. UV-Vis Spectroscopy: Detects changes in absorbance spectra, indicating effective biomolecule attachment;

4. Fourier Transform Infrared Spectroscopy (FTIR): Identifies functional groups and confirms covalent bonding;

5. X-ray Photoelectron Spectroscopy (XPS): Provides elemental composition and surface chemical modification details;

6. Scanning Electron Microscopy (SEM) and Transmission Electron Microscopy (TEM): Visualizes size, shape, and biomolecule attachment;

7. Scanning Probe Microscopy (SPM): Offers nanoscale imaging and quantitative analysis of surface modifications.

Cost-effectiveness of plasmonic metasurfaces for biosensing of infectious diseases

Plasmonic metasurfaces, particularly gold-based nanoparticles (AuNPs) have been employed as colorimetric and LSPR-based biosensors due to their high biocompatibility, sensitivity and selectivity, as well as high surface area-to-volume ratio, increasing the available space for the immobilization of biomolecules.[20,21] However, in addition to the inherent cost of the materials

used for the manufacturing of these devices, Table 1 in the main manuscript highlights that the fabrication methods that provide the best resolution are expensive, time consuming or labor-intensive, besides presenting a small device throughput. A template-based approach aims to overcome some of these limitations, reducing the dependence on lithography processes and facilitating the fabrication of metal-based metasurfaces for biodetection.[22]

Furthermore, recent applications of AuNPs have provided an increase in cost-effectiveness due to their advantages for biodetection of infectious diseases. For the detection of SARS-CoV-2, a 2D gold nano-islands-based chip has been employed, enhancing the real-time and label-free accuracy of the detection of different viral gene sequences.[21,23] In addition, colorimetric biosensing has been achieved with AuNPs capped with thiol-modified antisense oligonucleotides (AuNPs-ASOs), facilitating naked-eye detection of viral infectious diseases without involving sophisticated techniques, an important achievement for facilitating point-of-care diagnostic devices.[21,22,24]

For the detection of dengue virus (DENV), the use of AuNPs has achieved precise detection thresholds that serologically differentiate one of the four DENV serotypes from other flaviviruses, such as the zika virus (ZIKV) and the yellow fever virus (YFV), for a biosensor functionalized for one of the four DENV serotypes.[25,26] In addition, highly specific and sensitive magnetic relaxation nanosensors (MRnS) that operate based on surface plasmon resonance (SPR) have been developed to selectively identify ZIKV and cross-reactivity between flaviviruses that lead to false positives and inaccurate testing.[27]

The application of plasmonic metasurfaces for biosensing of other infectious diseases has also proven to be remarkably efficient. For instance, a pyramidal lattice achieved a sensibility 10^3 times higher than typical LSPR systems for the detection of paracoccidioidomycosis[22] and a nanostructure consisting of graphene and polythionine attached to gold nanorods (GNRs) as a biosensor for the human papillomavirus (HPV) DNA achieved a detection limit of 4.03×10^{-14} mol/l.[28] In addition, SERS applications of functionalized AuNPs have reported high sensitivity and specificity for visceral leishmaniasis (VL) for point-of-need diagnostics.[29]

References

(1) Ding, S.; Zhang, N.; Lyu, Z.; Zhu, W.; Chang, Y.; Hu, X. Protein-based nanomaterials and nanosystems for biomedical applications: A review. *Materials Today* **2021**, *43*, 166–184.

(2) Huang, Y.; Guo, X.; Wu, Y.; Chen, X.; Feng, L.; Xie, N.; Shen, G. Nanotechnology's frontier in combatting infectious and inflammatory diseases: prevention and treatment. *Signal Transduction and Targeted Therapy* **2024**, *9*.

(3) Jayakrishnan, A.; Rosli, W. R. W.; Tahir, A. R. M.; Razak, F. S. A.; Kee, P. E.; Ng, H. S.; Chew, Y.; Lee, S.; Ramasamy, M.; Tan, C. S.; Liew, K. B. Evolving Paradigms of Recombinant Protein Production in Pharmaceutical Industry: A Rigorous Review. *Sci* **2024**, *6*.

(4) Biffignandi, G. B.; Vola, A.; Sassera, D.; Najafi-Fard, S.; Morales, M. A. G.; Brunetti, E.; Teggi, A.; Goletti, D.; Petrone, L.; Tamarozzi, F. Antigen discovery by bioinformatics analysis and peptide microarray for the diagnosis of cystic echinococcosis. *PLOS Neglected Tropical Diseases* **2023**,

(5) Jr, J. C. A.; P, T.; M, W.; et al *Immunobiology: The Immune System in Health and Disease*; Garland Science, 2001.

(6) Haselbeck, A. H.; Im, J.; Prifti, K.; Marks, F.; Holm, M.; Zellweger, R. M. Serology as a Tool to Assess Infectious Disease Landscapes and Guide Public Health Policy. *Pathogens* **2022**, *11*.

(7) Zhou, J.; Chen, J.; Peng, Y.; Xie, Y.; Xiao, Y. A Promising Tool in Serological Diagnosis: Current Research Progress of Antigenic Epitopes in Infectious Diseases. *Pathogens* **2022**, *11*.

(8) Edén, A.; Grahn, A.; Bremell, D.; et al Viral Antigen and Inflammatory Biomarkers in Cerebrospinal Fluid in Patients With COVID-19 Infection and Neurologic Symptoms Compared With Control Participants Without Infection or Neurologic Symptoms. *JAMA Network Open* **2022**, *5*.

(9) Schuchardt, S.; Sickmann, A. *Plant Systems Biology*; Birkhäuser Basel, 2007.

(10) Noble, J. E.; Bailey, M. J. A. Chapter 8 Quantitation of Protein. *Methods in Enzymology* **2009**, *463*.

(11) He, F. Laemmli-SDS-PAGE. *Bio-protocol Exchange*

(12) Vallejo, L. F.; Rinas, U. Strategies for the recovery of active proteins through refolding of bacterial inclusion body proteins. *Microbial Cell Factories* **2004**, *3*.

(13) Tatkiewicz, W.; Elizondo, E.; Moreno, E.; Díez-Gil, C.; Ventosa, N.; Veciana, J.; Ratera, I. *Insoluble Proteins. Methods in Molecular Biology*; Humana Press, 2015; Vol. 1258.

(14) Greenfield, N. J. Using circular dichroism spectra to estimate protein secondary structure. *Nature Protocols* **2006**, *1*.

(15) Durowoju, I. B.; Bhandal, K. S.; Hu, J.; Carpick, B.; Kirkitadze, M. Differential Scanning Calorimetry — A Method for Assessing the Thermal Stability and Conformation of Protein Antigen. *JoVE Journal Biochemistry* **2017**,

(16) Huynh, K.; Partch, C. L. Analysis of Protein Stability and Ligand Interactions by Thermal Shift Assay. *Current Protocols in Protein Science* **2015**, *79*.

(17) Tabatabaei, M. S.; Ahmed, M. *Cancer Cell Biology. Methods in Molecular Biology*; Humana Press, 2022; Vol. 2508.

(18) Möller, M.; Denicola, A. Study of protein-ligand binding by fluorescence. *Biochemistry and Molecular Biology Education* **2006**, *30*, 309–312.

(19) Duff Jr., M. R.; Grubbs, J.; Howell, E. E. Isothermal Titration Calorimetry for Measuring Macromolecule-Ligand Affinity. *JoVE Journal Biology* **2011**,

(20) Fontana-Escartín, A.; Bertran, O.; Alemán, C. Materials engineering in electrochemical biosensors: A review of cost-effective approaches to efficient biodetection. *Materials Today Communications* **2024**, *41*, 111030.

(21) Perveen, S.; Negi, A.; Gopalakrishnan, V.; Panda, S.; Sharma, V.; Sharma, R. COVID-19 diagnostics: Molecular biology to nanomaterials. *Clinica Chimica Acta* **2023**, *538*, 139–156.

(22) Marques, T. E. M. et al. Tunable Surface Plasmon-Polaritons Interaction in All-Metal Pyramidal Metasurfaces: Unveiling Principles and Significance for Biosensing Applications. *ACS Applied Optical Materials* **2024**, *2*, 1374–1381.

(23) Qiu, G.; Gai, Z.; Tao, Y.; Schmitt, J.; Kullak-Ublick, G. A.; Wang, J. Dual-Functional Plasmonic Photothermal Biosensors for Highly Accurate Severe Acute Respiratory Syndrome Coronavirus 2 Detection. *ACS Nano* **2020**, *14*, 5268–5277.

(24) Moitra, P.; Alafeef, M.; Dighe, K.; Frieman, M. B.; Pan, D. Selective Naked-Eye Detection of SARS-CoV-2 Mediated by N Gene Targeted Antisense Oligonucleotide Capped Plasmonic Nanoparticles. *ACS Nano* **2020**, *14*, 7617–7627.

(25) Versiani, A. F.; Martins, E. M. N.; Andrade, L. M.; Cox, L.; Pereira, G. C.; Barbosa-Stancioli, E. F.; Nogueira, M. L.; Ladeira, L. O.; da Fonseca, F. G. Nanosensors based on LSPR are able to serologically differentiate dengue from Zika infections. *Scientific Reports* **2020**, *10*.

(26) Machado, G. L.; Teixeira, F. M. F.; Ferreira, G. S. C.; Versiani, A. F.; Andrade, L. M.; Ladeira, L. O.; da Fonseca, F. G.; Ramirez, J. C. Computational Guided Method Applied to LSPR-Based Biosensor for Specific Detection of the Four-Serotypes of Dengue

Virus in Seropositive Patients. *Particle & Particle Systems Characterization* **2022**, *39*, 2100157.

(27) Banerjee, T.; Patel, T.; Pashchenko, O.; Elliott, R.; Santra, S. Rapid Detection and One-Step Differentiation of Cross-Reactivity Between Zika and Dengue Virus Using Functional Magnetic Nanosensors. *ACS Applied Bio Materials* **2020**, *4*, 3786–3795.

(28) Versiani, A. F.; Andrade, L. M.; Martins, E. M.; Scalzo, S.; Geraldo, J. M.; Chaves, C. R.; Ferreira, D. C.; Ladeira, M.; Guatimosim, S.; Ladeira, L. O.; da Fonseca, F. G. Gold Nanoparticles and Their Applications in Biomedicine. *Future Virology* **2016**, *11*, 293–309.

(29) Mancini, R. S. N.; Sabaine, A. E.; Castro, C. E.; Carnielli, J. B. T.; Dietze, R.; de Oliveira, V. L.; Lanfredi, A. J. C.; Kubota, L. T.; Mamián-López, M. B.; Alves, W. A. Development and Validation of a SERS-Based Serological Test Combined with PLS-DA Method for Leishmaniasis Detection. *ACS Applied Electronic Materials* **2022**, *4*, 3997–4006.
